# Management of elderly patients with a non-ST-segment-elevation acute coronary syndrome

**DOI:** 10.1007/s12471-017-1002-3

**Published:** 2017-05-17

**Authors:** M. E. Gimbel, J. M. ten Berg

**Affiliations:** Department of Cardiology, St. Antonius Hospital, Nieuwegein, The Netherlands

**Keywords:** Acute coronary syndrome, Aged, Percutaneous coronary intervention, Platelet aggregation inhibitors, Coronary angiography, Elderly

## Abstract

Elderly patients with an acute coronary syndrome are underrepresented in randomised controlled trials. Neither the European Society of Cardiology nor the American Heart Association/American College of Cardiology acute coronary syndrome guidelines provide specific recommendations for elderly patients. However, elderly patients are at higher thrombotic and bleeding risk compared with younger patients leading to difficulties in choosing the optimal treatment. In this review, we discuss the uncertainties we encounter in treating elderly patients with non-ST-elevation acute coronary syndrome and suggest treatment options based on the existing literature.

## Introduction

Elderly patients portray a heterogeneous group due to their comorbidities and differences in cognition and functional status. They comprise a rapidly increasing subgroup of patients with an acute coronary syndrome (ACS) of which non-ST-segment-elevation ACS (NSTE-ACS) is the most common form [1]. Compared with younger patients, elderly patients with ACS are at higher risk of both atherothrombotic events and bleeding, due to frailty and comorbidities such as renal failure [2–4]. These higher risks require a different treatment. However, neither the European Society of Cardiology (ESC) [5] nor American Heart Association/American College of Cardiology (AHA/ACC) [6] ACS guidelines provide specific recommendations regarding the treatment of elderly patients. In addition, most evidence is expert consensus or based on subgroup analyses of randomised controlled trials (RCTs) in which the elderly were underrepresented. This complicates every day clinical decision-making regarding the optimal treatment of these elderly patients.

In this article, we discuss the uncertainties we encounter in treating elderly patients with NSTE-ACS and suggest treatment options based on the existing literature. Our search is shown in the Appendix.

## Step one: Risk assessment

The first step in treating ACS patients is assessing ischaemic and bleeding risk for which the guidelines recommend the use of the GRACE and CRUSADE risk scores [2, 3]. Following the guidelines, an ACS patient with a higher ischaemic risk would require strong-acting antiplatelet inhibitors and an invasive strategy, while in patients with a higher bleeding risk one would be cautious with an invasive strategy and preference could be given to less potent antiplatelet inhibitors. However, there is much overlap between risk factors indicating ischaemic and bleeding complications (Tables 1 and 2). Consequently, a considerable number of elderly patients have both a high predicted ischaemic and bleeding risk. Thus, these risk scores are not very helpful in determining the optimal antithrombotic treatment. In addition, it is questionable whether the risk scores are as predictive in the elderly as in younger patients. Research on the applicability of the GRACE score in 544 elderly (≥80 years) NSTE-ACS patients showed good diagnostic accuracy for the prediction of in-hospital mortality with an area under the curve (AUC) of 0.75 [7], while the CRUSADE bleeding score was not predictive in 369 elderly patients ≥75 years as compared with 1667 younger patients <75 years (AUC 0.52 vs. AUC 0.74) [8]. Other bleeding scores (ACTION and Mehran) were not predictive in elderly patients either [8].

**Table 1:** GRACE risk score

Risk factor at admission
Heart rate (bpm)^a^
Systolic blood pressure (mm Hg)^a^
Creatinine (µmol/l)^a^
Chronic heart failure^a^
Elevated cardiac enzymes/markers
Age (years)
Cardiac arrest at admission
ST-segment deviation

*Bpm* beats per minute

^a^Risk factors indicating ischaemic and bleeding complications

**Table 2:** CRUSADE bleeding score

Risk factor at admission
Heart rate (bpm)^a^
Systolic blood pressure (mm Hg)^a^
GFR: Cockcroft-Gault^a^
Chronic heart failure^a^
Prior vascular disease
Diabetes mellitus
Baseline haematocrit (%)
Sex

*Bpm* beats per minute, *GFR* glomerular filtration rate

^a^Risk factors indicating ischaemic and bleeding complications

Based on these data, we advise to use the GRACE score in elderly patients, whereby a high score is an indication for strong-acting platelet inhibitors and an invasive strategy. To address modifiable bleeding risk factors we advise using the CRUSADE score. Only in those patients with the highest risk of bleeding (i. e. prior history of bleeding, cerebrovascular accident, oral anticoagulant usage, frailty and malignancy) do we advise to use less potent antiplatelet agents. In addition, caution with an invasive therapy is advised in the frail elderly.

## Step two: Measures to reduce bleeding risk

Bleeding risk can be reduced by taking some treatment options into account. First, to reduce gastrointestinal bleeding risk, the guidelines advise to prescribe a proton-pomp inhibitor (PPI) in patients with prior gastrointestinal ulcer or haemorrhage, oral anticoagulant therapy, chronic NSAID/corticosteroid use or two or more of the following: age ≥65 years, dyspepsia, gastroesophageal reflux, *Helicobacter pylori* infection and chronic alcohol use. The prevention of gastrointestinal ulcer or haemorrhage with a PPI has been proven in a randomised trial with 991 patients aged ≥60 years receiving aspirin without gastric or duodenal ulcer at baseline endoscopy who were treated with esomeprazole or placebo. Esomeprazole reduced the risk of gastroduodenal ulcers in comparison with placebo (4.4% vs 18.3%, *p* < 0.0001) after 6 months [9]. Thus, more liberal to what is advised in the guidelines, it may be beneficial to treat all patients ≥60 years using aspirin with a PPI.

Second, use the transradial access when performing coronary angiography. A meta-analysis consisting of 24 randomised trials and 22,843 participants compared radial versus femoral access for catheterisation and found a reduction in major bleeding (OR 0.71 [95%CI 0.48–1.04]) and major vascular complications (OR 0.26 [95% CI 0.17–0.41]) when a radial approach was used [10]. One of the included trials was The RadIal Versus femorAL access for coronary intervention (RIVAL) trial which randomised 7021 ACS patients, including 1035 (15%) over the age of 75 years, to radial or femoral access. The elderly subgroup analysis demonstrated a superior effect of transradial access driven by a reduction in major access site complications of 3.6% vs 6.6% *p* = 0.03 [11]. However, when the radial approach fails, the femoral approach with bivalirudin has proven to yield similar bleeding rates compared with radial access and using heparin in a meta-analysis of eight randomised trials including 27,491 ACS patients [12].

Third, in patients undergoing percutaneous coronary intervention (PCI), fondaparinux with a bolus of unfractionated heparin (UFH) is advised rather than enoxaparin by both the ESC guidelines for ACS and the expert position paper on antithrombotic therapy in the elderly [5, 13]. This is based on the Organization to Assess Strategies in Acute Ischemic Syndromes (OASIS)-5 trial which revealed a net clinical benefit (death, myocardial infarction (MI), refractory ischaemia or major bleeding) in favour of fondaparinux (7.3% vs 9.0%, HR 0.81, *p* < 0.001) compared with enoxaparin which was consistent in the subgroup of 12,261 (61%) patients ≥65 years [14]. Caution is advised with the use of enoxaparin in the elderly as the Superior Yield of the New Strategy of Enoxaparin, Revascularization, and GlYcoprotein IIb/IIIa inhibitors (SYNERGY) trial, comparing enoxaparin versus UFH, observed a higher, although nonsignificant (p = 0.085) bleeding risk with enoxaparin compared with UFH in 2540 (25%) elderly patients (≥75 years) [15]. Therefore UFH might be the anticoagulant of choice in elderly patients who cannot receive fondaparinux. However, also bivalirudin could be second choice as beneficial results were observed in the Randomized Evaluation in PCI Linking Angiomax to reduced Clinical Events (REPLACE)-2 trial, determining the efficacy of bivalirudin with bailout glycoprotein IIb/IIIa inhibition compared with heparin with planned glycoprotein IIb/IIIa inhibition. Bivalirudin significantly reduced the rates of in-hospital major bleeding by 2.4% vs 4.1%, *p* < 0.001. A subgroup analysis of 806 (13.4%) elderly patients (>75 years) [16] showed the same results.

## Step three: Antiplatelet therapy

According to the guidelines, treatment of NSTE-ACS patients consists of lifelong aspirin and a P2Y_12_ inhibitor for one year. What evidence is there to treat elderly patients with aspirin?

Aspirin reduced death or MI by 48% after 12 months in a placebo-controlled randomised trial including 796 ACS patients [17]. This effect was confirmed by a meta-analysis of 96,316 patients performed by the Antiplatelet Trialists’ Collaboration. Moreover, the benefit of aspirin seems even greater in older compared with younger patients [18]. Therefore aspirin is advised in all elderly ACS patients except for those with severe renal and liver insufficiency, severe uncontrolled heart failure or an active peptic ulcer.

The preferred P2Y_12_ inhibitor is ticagrelor, or prasugrel in those patients in whom the coronary anatomy is known and who are going to proceed to PCI. Do these recommendations also apply to the elderly?

The PLATelet inhibition and patient Outcomes (PLATO) trial randomised 18,624 ACS patients to ticagrelor or clopidogrel on top of aspirin and found a significant clinical benefit and overall safety for ticagrelor (death from vascular causes, MI or stroke 9.8% vs 11.7%; TIMI major bleeding 7.9% vs 7.7%). This beneficial effect also seems true for the elderly (≥75 years) as the advantages of ticagrelor compared with clopidogrel were similar in the elderly subgroup analysis (cardiovascular death, MI or stroke 17.2% vs 18.3%; overall PLATO major bleeding 14.2% vs 13.5%) [19, 20].

The Trial to Assess Improvement in Therapeutic Outcomes by Optimizing Platelet Inhibition with Prasugrel – Thrombolysis in Myocardial Infarction (TRITON-TIMI) 38 randomly assigned 13,608 patients to prasugrel or clopidogrel. Prasugrel reduced the risk of death from cardiovascular causes, MI or stroke by 2.2% (9.9% vs 12.1%); however, non-CABG related TIMI major bleeding was significantly higher (2.4% vs 1.8%). A post-hoc analysis identified elderly patients aged ≥75 years as deriving no net-clinical benefit for prasugrel compared with clopidogrel 0.99 (95% CI 0.81–1.21; *p* = 0.92) [21] driven by higher rates of bleeding in the elderly, where fatal bleeding occurred in 1% of the patients treated with prasugrel versus 0.1% in those treated with clopidogrel [21]. Consequently, prasugrel 10 mg is not recommended for elderly patients ≥75 years.

However, a pharmacokinetic modelling substudy from TRITON-TIMI 38 predicted that a 5 mg prasugrel exposure in a subgroup of 891 elderly (≥75 years) patients would reduce bleeding risk and maintain efficacy [22]. This was further evaluated in The Comparison of Prasugrel and Clopidogrel in Very Elderly and Non-Elderly Patients With Stable Coronary Artery Disease (GENERATIONS) trial which examined the pharmacokinetic and pharmacodynamic response of prasugrel 5 mg in elderly patients (≥75 years) and found non-inferiority of the reduced dose in the elderly compared with the 10 mg dose in non-elderly. Moreover, prasugrel 5 mg compared with clopidogrel 75 mg in elderly patients was associated with a significantly greater antiplatelet effect [23]. In the Targeted Platelet Inhibition to Clarify the Optimal Strategy to Medically Manage Acute Coronary Syndromes (TRILOGY-ACS) trial medically managed ACS patients were randomised to prasugrel or clopidogrel [24]. Of the 9326 included patients, 2083 were ≥75 years and were treated with prasugrel 5 mg. This reduced dose of prasugrel in elderly patients was non-inferior to clopidogrel in preventing ischaemic complications and no differences in bleeding rates were observed [25]. Consequently, if prasugrel is used in the treatment of elderly ACS patients, the reduced dosage of 5 mg is recommended.

Clopidogrel is advised in patients who cannot receive ticagrelor or prasugrel or require oral anticoagulation. The Clopidogrel in Unstable Angina to Prevent Recurrent Events (CURE) trial investigated the effect of clopidogrel versus placebo in addition to aspirin in 12,652 patients with NSTE-ACS. CURE showed a significant 20% reduction in the composite outcome of cardiovascular death, nonfatal MI or stroke in patients receiving clopidogrel [26]. An equal benefit of clopidogrel was observed in young and elderly patients (≤65 vs >65 years). Recently, an observational study including 190 patients aged ≥75 years admitted for MI identified frailty as an independent predictor of major bleeding [4]. Therefore, in addition to patients who cannot receive ticagrelor or prasugrel, we advise to also treat the frail elderly patients with clopidogrel. Currently, the Ticagrelor or prasugrel versus clopidogrel in elderly patients with an acute coronary syndrome and a high bleeding risk: optimization of antiplatelet treatment in high-risk elderly (POPular AGE) trial is investigating whether elderly (≥70 years) NSTE-ACS patients should be treated with ticagrelor/prasugrel or clopidogrel using a randomised controlled design [27].

Furthermore, PRAGUE-18 performed a head-to-head comparison between ticagrelor and prasugrel. A total of 121 (9.8%) elderly patients (≥75 years) were included [28]. There were no differences in safety and efficacy between ticagrelor and prasugrel; this was consistent in the elderly. However, an interim analysis after 1130 included patients led to the decision to terminate the study early because of futility.

## Step four: Timing of the P2Y_12_ inhibitor administration

The guidelines advise not to administer prasugrel before coronary angiography based on the Comparison of Prasugrel at the Time of PCI or as Pretreatment at the Time of Diagnosis in Patients with Non-ST Elevation Myocardial Infarction (ACCOAST) which showed no reduction in major ischaemic events in NSTE-ACS patients scheduled for catheterisation and pretreated with prasugrel compared with placebo [29]. TIMI major bleeding was significantly more frequent in the prasugrel group. These findings were consistent in 715 (18%) elderly patients aged ≥75 years. Pretreatment with ticagrelor or clopidogrel has not been adequately investigated and therefore no recommendations are formulated in the guidelines. The PCI-CURE showed a reduction in cardiovascular death or MI by about a third with pretreatment with clopidogrel compared with placebo, with little difference in bleeding rate [30]. There is no specific research regarding pretreatment with ticagrelor in NSTE-ACS patients. However, a subgroup analysis from PLATO showed equal benefit of dual antiplatelet therapy (DAPT) with ticagrelor in patients intended for non-invasive management [31]. Therefore we consider pretreatment with clopidogrel and ticagrelor safe when other diagnoses such as aortic dissection are unlikely or ruled out. Yet, in elderly patients at a high risk of bleeding we advise to postpone the administration of the P2Y_12_ inhibitor until after angiography.

## Step five: Invasive management

The guidelines recommend an invasive strategy in addition to the antithrombotic treatment in most ACS patients. Does this also apply to the elderly?

Compared with younger ACS patients, the elderly are less likely to undergo coronary angiography and subsequent revascularisation [32, 33]. However, real-world data from registries showed benefit from an invasive strategy, particularly in the elderly. Indeed, this effect seems to increase with increasing age [33, 34]. A meta-analysis comparing the routine invasive strategy with selective invasive strategy in NSTE-ACS patients reported a lower hazard of cardiovascular death or MI in patients ≥65 years undergoing invasive therapy (26.1% vs 34.9%, *p* = 0.007) [35]. Also, the Treat Angina with Aggrastat and Determine Cost of Therapy with an Invasive or Conservative Strategy – Thrombolysis in Myocardial Infarction (TACTICS-TIMI) 18 trial found a substantial reduction in death or MI at 6 months in the elderly patient (≥75 years) undergoing invasive strategy compared with conservative strategy, mainly driven by a reduction in non-fatal MI (21.6% vs 10.8%). However, the invasive strategy led to significantly higher rates of major bleeding in older patients (16.6% vs 6.5%) compared with the conservative strategy [36]. This was consistent in the Global Registry of Acute Coronary Events (GRACE) registry where the rates of major bleeding according to age were <70 years: 2.2%; 70–80 years: 3.3%; >80 years: 7.0% [37]. The Italian Elderly ACS trial found no significant differences in death, MI or severe bleeding between invasive versus initially conservative treatment in 313 elderly patients (≥75 years) with NSTE-ACS. Although, a subgroup analysis showed significant benefit from invasive treatment in patients with elevated troponin levels [38]. Also the coMOrbilidades en el Síndrome Coronario Agudo (MOSCA) study found no significant differences between invasive versus conservative treatment in comorbid elderly patients (≥70 years) with non-ST-segment-elevation MI [39]. Both trials were ended prematurely. The After Eighty study provides the best evidence in favour of a routine invasive strategy in 457 clinically stable elderly patients (≥80 years) with NSTE-ACS and low bleeding rates (1.7% vs 1.8%) [40]. The low bleeding rates could be due to the predominant use of the radial artery and selection of clinically stable elderly patients. Attenuation of the efficacy of an invasive strategy was observed with increasing age. Furthermore, generalisability to all elderly patients is debatable as only 457 of the 4187 octogenarians with NSTE-ACS were included.

The ESC guideline recommends timing of invasive strategy based on risk criteria for ischaemic complications. The immediate invasive strategy is restricted to haemodynamically unstable patients and patients with angina while on maximal medical treatment. Patients with a significant rise or fall in cardiac troponin, dynamic ECG or GRACE score >140 should undergo angiography within 24 h. Patients with diabetes mellitus, renal insufficiency, decreased left ventricular ejection fraction or congestive heart failure, early post-infarction angina, prior PCI or CABG and GRACE risk score >109 to <140 should undergo angiography within 72 h. Reviewing the literature, advantage of early versus delayed invasive strategy was driven by a reduction in episodes of refractory ischaemia and shorter hospital stay; these results were consistent in elderly subgroup analyses [41–43].

We advise, in accordance with the guidelines, to treat the elderly high-risk NSTE-ACS patients with a routine invasive strategy. However, as frailty is associated with higher rates of adverse long-term outcomes after PCI [44], clinical assessment of frailty and expected benefit of the procedure should always precede the decision to perform coronary angiography.

## Step six: Duration of dual antiplatelet therapy

Controversy exists about the optimal duration of DAPT after NSTE-ACS. The guidelines recommend DAPT for one year, but based on individual ischaemic and bleeding risk, duration of DAPT may be shortened (3–6 months) or extended (up to 30 months).

The Prevention of Cardiovascular Events in Patients with Prior Heart Attack using Ticagrelor Compared with Placebo on a Background of Aspirin – Thrombolysis in Myocardial Infarction (PEGASUS-TIMI) 54 trial investigated the effect of prolonged DAPT with ticagrelor in patients with prior MI [45]. Ticagrelor 60 mg was superior in reducing cardiovascular death, MI or stroke over placebo in 3083 (15%) patients aged ≥75 years (11.0% vs 13.9% vs 13.5% respectively). However, elderly patients derived more harm from ticagrelor 60 mg compared with placebo (TIMI major bleeding: 4.11% vs 1.68%). Furthermore, patients at high bleeding risk (i. e. prior history of stroke, known bleeding disorder or recent bleeding history) were excluded from the study.

A meta-analysis, comparing extended DAPT versus aspirin alone in patients with prior MI found an overall relative risk (RR) of major adverse cardiovascular events of 0.78 in favour of prolonged DAPT; this was consistent in the elderly (≥75 years). However, bleeding risk was increased (RR 1.92) although the rate of fatal bleeding and intracerebral haemorrhage was not significantly different. Furthermore, there was a trend towards reduced all-cause mortality [46]. Another meta-analysis comparing short-term DAPT (3–6 months) with 12-months of DAPT in low-risk patients with mainly stable coronary artery disease and drug-eluting-stents showed a significant reduction in major bleeding (OR 0.58) with short-term DAPT with no significant differences in ischaemic events. This was consistent in patients aged ≥65 years [47].

Recent trials (Zotarolimus-Eluting versus Bare-Metal Stents in uncertain Drug-Eluting Stent Candidates (ZEUS)) [48] and Prospective Randomized Comparison of the BioFreedom Biolimus A9 Drug-Coated Stent versus the Gazelle Bare-Metal Stent in Patients at High Bleeding Risk (LEADERS-FREE) [49] supported a DAPT duration of one month after second generation drug-eluting stent implantation in patients at high bleeding risk or unable to use DAPT for a longer time period. A consistent treatment effect was observed in the elderly (≥75 years) [49].

Taking the above into consideration, we would advise to treat elderly NSTE-ACS patients with DAPT for 12 months. In non-frail, elderly patients without any bleeding during the first year of DAPT and at high ischaemic risk one could consider extended DAPT duration. In elderly patients at high bleeding risk, we recommend a shorter DAPT duration (3–6 months), although, scientific evidence is scarce.

## Conclusion

This article provides an overview on the optimal management of elderly patients with NSTE-ACS based on the existing literature. For most elderly NSTE-ACS patients, treatment should consist of DAPT with aspirin and ticagrelor given at the time of diagnosis together with fondaparinux. A routine invasive approach is preferred using transradial access and a bolus of UFH. Probably all elderly patients treated for NSTE-ACS should start with a PPI. DAPT for 12 months seems to be the most optimal duration in most elderly patients. Furthermore, it is important to assess frailty rather than age when considering the optimal treatment. However, there is still much debate about the optimal treatment in these elderly patients as most evidence is based on subgroup analysis and trials with an unrepresentative participation of elderly patients. Further research is needed to increase certainty about treatment strategies in elderly ACS patients.

## Appendix

We used references from the ESC and AHA/ACC guidelines and the expert position paper of Andreotti et al. on anti-thrombotic therapy. Furthermore, we hand searched PubMed using synonyms for elderly (aged [Mesh] OR elder*[tiab] OR octogenarian*[tiab] OR nonagenarian*[tiab] OR centenarian*[tiab]) and non-ST-elevation myocardial infarction (non-ST elevated myocardial infarction[Mesh] OR angina, unstable [Mesh] OR unstable angina[tiab] OR non-ST-elevat*[tiab] OR non-ST-segment elevat*[tiab] OR without ST-segment elevat*[tiab] OR non-STEMI[tiab] OR NSTEMI[tiab]) and coronary angiography (percutaneous coronary intervention [Mesh] OR coronary angiography [Mesh] OR percutaneous coronary intervention*[tiab] OR angiograph*[tiab] OR cardiac catheter*[tiab]) and antiplatelet therapy (ticagrelor[tiab] OR clopidogrel[tiab] OR prasugrel[tiab]). We also assessed the references from the selected articles.
